# Reply to Saumitou‐Laprade et al. (2017) “Controlling for genetic identity of varieties, pollen contamination and stigma receptivity is essential to characterize the self‐incompatibility system of *Olea europaea* L.”. Eva:https://doi.org/10.1111/eva.12498

**DOI:** 10.1111/eva.12633

**Published:** 2018-04-17

**Authors:** Daniela Farinelli, Catherine Breton, Georgios Koubouris, Franco Famiani, Pierre Villemur, André Bervillé

**Affiliations:** ^1^ Dipartimento di Scienze Agrarie Alimentari e Ambientali (DSA3) Università degli Studi di Perugia Perugia Italy; ^2^ Institut des Sciences de l’Évolution de Montpellier (ISE‐M) UMR CNRS 5554 Montpellier France; ^3^ Institute for Olive Tree, Subtropical Crops & Viticulture Hellenic Agricultural Organization “Demeter” (ex. NAGREF) Chania Greece; ^4^ SupAgro, 34060 Montpellier Cedex France; ^5^ INRA, UMR‐DIAPC 1097 Montpellier France

**Keywords:** fruit setting calculation, genetic identity, morphological characterization, paper bag, pollen contamination, pollen germination test, stigma receptivity

## Abstract

This study was carried out to examine the validity of previous studies on the intercompatibility of olive and to compare the approach and techniques used for proposing the diallelic self‐incompatibility system and the sporophytic self‐incompatibility system. Analysis of the literature indicates that the mating system of the olive tree is a controversial issue and requires further studies to clearly and fully comprehend it. All possible approaches should be used to maximize reliability of the final conclusions on the olive mating system.

## 1 INTRODUCTION

Most of the studies on the intercompatibility of olive varieties carried out in recent decades have been reported to be debatable (Saumitou‐Laprade, Vernet, Vekemans, Billiard et al., 2017; Saumitou‐Laprade, Vernet, Vekemans, Castric et al., 2017), because of (i) “the vast uncertainty around the genetic identity of vernacular varieties,” (ii) “the massive risk of contamination associated with commonly used pollination protocols,” and (iii) proper attention not given to stigma receptivity. Moreover, on the basis of new data showing no asymmetry on the varieties they used, Saumitou‐Laprade, Vernet, Vekemans, Castric et al. (2017) claimed that discrepancies with cases of asymmetry asserted in previous studies were due to the above reported three factors and so they expressed concern about the sporophytic self‐incompatibility (SSI) system proposed by Breton et al. (2014). The aim of this study was to examine more thoroughly the validity of the previous studies on olive intercompatibility and self‐compatibility (Al‐Kasasbeh, Atteyeh, & Qrunfleh, 2005; Androulakis & Loupassaki, 1990; Ateyyeh, Stosser, & Qrunfleh, 2000; Bradley, Griggs, & Hartmann, 1961; Cuevas & Polito, 1997; Cuevas et al., 2001; Dimassi, Thermos, & Balatsos, 1999; Eassa, El‐Tweel, & Gorda, 2011; El‐Hady, Haggag, Abdel‐Migeed, & Desouky, 2007; Farinelli, Boco, & Tombesi, 2006; Farinelli, Hassani, & Tombesi, 2008b; Fernandez‐Escobar & Gomez–Valledor, 1985; Griggs, Hartmann, Bradley, Iwakiri, & Whisler, 1975; Iannotta, Briccoli Bati, Perri, & Tocci, 1999; Koubouris, Breton, Metzidakis, & Vasilakakis, 2014; Lavee & Datt, 1978; Morettini, Bini, & Bellini, 1972; Moutier, 2002; Moutier, Garcia, Féral, & Salles, 2001; Seifi, Guerina, Kaiser, & Sedgley, 2011; Sharma, Thakur, & Sharm, 1976; Spinardi & Bassi, 2012; Taslimpour, Bonyampour, & Rahemi, 2008; Tombesi, 1978; Tombesi, Cartechini, & Preziosi, 1982; Vuletin Selak, Perica, Goreta Ban, Radunic, & Poljak, 2011), and to analyze the techniques and data used by (Saumitou‐Laprade, Vernet, Vekemans, Billiard et al. (2017); Saumitou‐Laprade, Vernet, Vekemans, Castric et al. (2017)), which are at the basis of the concern about the SSI system proposed by Breton et al. (2014).

## 2 CONTROLLING FOR THE GENETIC IDENTITY OF VARIETIES

Most of the studies regarding the genetic identification of different varieties of olive and other fruit species have been performed using morphological characteristics. The statement “vast uncertainty around the genetic identity of vernacular varieties” seems exaggerated because there could be uncertainty in some specific situations, but not as a general case, especially for widely grown cultivars. In the case of olive, the morphological characteristics used to identify cultivars are those that have been widely used for descriptive purposes, and for distinguishing and classifying olive cultivars (Pandolfi et al., 2004; Barranco & Rallo, 1985, 2000; Barranco et al., 2000; Bartolini, Prevost, Messeri, & Carignani, 2008; Bartolini, Prevost, Messeri, Carignani, & Menini, 1998; Cantini, Cimato, & Sani, 1999; Cimato, Cantini, & Sani, 2001; Cimato, Cantini, Sani, & Marranci, 1997; del Río & Caballero, 1994; Lombardo, 2003; Pannelli, Alfei, D'Ambrosio, Rosati, & Famiani, 2000; Pannelli, Alfei, & Santinelli, 1998; Rotondi, Magli, Ricciolini, & Baldoni, 2003). The validity of using morphological characteristics to recognize olive varieties is supported by the fact that a methodology has been developed for “morphological characterization.” Furthermore, this procedure is at the basis of the protocol of the Community Plant Variety Office (CPVO), which is the office that describes the technical procedures to be followed in order to meet Council Regulation (EC) N°2100/94 on Community Plant Variety Rights. For olive, procedures are based on UPOV Document TG/1/3 and UPOV Guideline TG/99/4 dated 20/10/2011 for conducting tests for distinctness, uniformity, and stability of varieties (Community Plant Variety Office (CPVO), 2012). Moreover, morphological characterization, with or without molecular characterization, is at the basis of the controls for variety correspondence for the registration of primary sources in the certification process of plants produced by nurseries (for which the identity of the cultivar is essential) (Baldoni et al., 2011a,b). In addition, the overall validity of using morphological characteristics to recognize cultivars is also demonstrated by the fact that the identity of the majority of varieties classified by examining morphological characteristics (Farinelli et al., 2006, 2008b) has also been confirmed by genetic characterization (Mousavi et al., 2017). This furtherly confirms that errors in identifying cultivars using morphological characterization only represent exceptions and not the rule.

## 3 REGARDING POLLEN CONTAMINATION

Obviously, some minor pollen contamination is likely to occur even when precautionary measures are taken. Referring to De La Rosa, James, and Tobutt (2004), Saumitou‐Laprade, Vernet, Vekemans, Castric et al. (2017) wrote that massive contamination was demonstrated in cultivar crosses, with as many as 96 of 149 (64 %) of the progenies, whose expected father could be genetically excluded, indicating that this was due to pollen contamination. However, in another study (Diaz, Martín, Rallo, & De la Rosa, 2007), only 17 % of the progenies showed that the expected father could be genetically excluded, indicating variable results. It is important to point out that De La Rosa et al. (2004) and Diaz et al. (2007) used different types of bags with respect to those used in other studies. In particular, De La Rosa et al. (2004) used double perforated plastic bags to cover the branches of the trees used as female parents, whereas in most other studies, such as Villemur, Musho, Delmas, Maamar, and Ouksili (1984), Farinelli et al. (2006, 2008b), Seifi et al. (2011), and Spinardi and Bassi (2012), paper bags were used. The different materials of which bags are made are important because of their possible permeability to pollen grains. The use of two bags of micropore paper or one bag of brown matte paper proved to ensure good impermeability to pollen and therefore no substantial contamination (del Río & Caballero, 1999). This is verified by the fact that using paper bags, self‐pollinated flowers showed no fruit or very low amounts for self‐incompatible cultivars (Farinelli et al., 2006, 2008b; Methamem, Gouta, Mougou, Bayoudh, & Boujnah, 2015; Spinardi & Bassi, 2012; Vuletin Selak et al., 2011). This is also reinforced by the observation that in long‐term studies, self‐incompatible cultivars gave similar results in all the years (Methamem et al., 2015; Shemer et al., 2014; Vuletin Selak et al., 2011). Furthermore, when the same self‐incompatible cultivar, such as Leccino, which is a widespread variety in Italy, was tested with paper bags in several environments, self‐incompatibility was always observed (Farinelli et al., 2006; Spinardi & Bassi, 2012; Vuletin Selak et al., 2011). In order to improve the accuracy of the bagging technique, it is important to bag the branches before the anthesis of all the cultivars in the field, and this was normally done when using this methodology (Farinelli et al., 2006). This minimizes the amount of pollen in the air, and thus the risk of contamination at the time of bagging (Cuevas & Polito, 1997; Diaz, Martin, Rallo, Barranco, & De la Rosa, 2006).

Obviously, in cross‐pollination treatments, contamination might occur during pollen transfer when the bags are opened. However, in order to quantify the incidence of unwanted pollen, it is interesting to examine data by Moutier, Terrien, Pécout, Hostalnou, and Margier (2006), who used male‐sterile varieties. They evaluated the rate of pollen contamination by opening and reclosing the bag placed before flowering, at the time of stigma receptivity—the duration of which is 4–8 days depending on the variety (Villemur et al., 1984). They found that only one of eight bags showed fruits, which were not expected. Is this rate 1/8 (12.5%) high enough to deny all studies based on bags? Indeed, this does not indicate “massive contamination.” In the studies carried out with this method, if massive pollen contamination had occurred, abundant cross‐pollination would have caused relatively high fruit setting rates in all the treatments studied, and this was definitely not the case (Farinelli et al., 2006; Spinardi & Bassi, 2012; Vuletin Selak et al., 2011). Furthermore, there would not have been different responses between the different crosses, as were reported in several studies (Cuevas & Polito, 1997; Farinelli et al., 2006; Shemer et al., 2014).

The validity of the use of correct bags and times for bagging and, more in general, of the bagging method if properly applied, is also demonstrated by the statistically significant results regarding the influence of the pollinizer on seed characteristics of the olives obtained. If massive contamination had occurred, significant effects on seed characteristics would not have been found as a result of characters transmitted by the male pollen, as instead reported by Cuevas and Oller (2000), Farinelli, Hassani, and Tombesi (2008a), and Farinelli, Pierantozzi, and Palese (2012).

## 4 STIGMA RECEPTIVITY

Stigma receptivity is essential to characterize the “self‐incompatibility system” of the olive tree (Rallo, Cuevas, & Rapoport, 1990). Villemur et al. (1984) stressed the importance of stigma receptivity. On average, stigma receptivity lasts for about a week (Tombesi et al., 1982; Villemur et al., 1984). In this regard, it has to be noted that for one branch under the bag, there is a mixture of flowers at different stages for each inflorescence and thus the total absence of receptivity cannot explain the failure of a cross, when pollen is added. Only very few varieties, such as Lucques, may have a shorter period of receptivity, about 4 days. In this case, in order to reduce the problems related to stigma receptivity, flowers of the pollinizer variety should be inserted in the bag twice during anthesis to ensure that abundant fresh pollen is available during stigma receptivity of the varieties studied (Koubouris et al., 2014). In any case, some fertilization should be ensured by the fact that in the bags there is a mixture of flowers at different stages and that the pollen inserted for artificial pollination remains in the bag. Therefore, in some cases fruit set could be underestimated, but, if a proper number of repetitions are used, the main results about compatibility should remain.

## 5 COMPARING RESULTS OF (SAUMITOU‐LAPRADE, VERNET, VEKEMANS, BILLIARD ET AL. (2017); SAUMITOU‐LAPRADE, VERNET, VEKEMANS, CASTRIC ET AL. (2017)) WITH OTHER STUDIES

Saumitou‐Laprade, Vernet, Vekemans, Billiard et al. (2017) Saumitou‐Laprade, Vernet, Vekemans, Castric et al. (2017) reported results on self‐incompatibility and divided olive cultivars into two groups, namely G1 and G2, which were not intercompatible within each group and were intercompatible between the two groups and asserted no asymmetry in reciprocal crosses. They based their conclusions especially on reciprocal stigma tests and pollen germination analysis. Using these findings, they proposed the diallelic self‐incompatibility (DSI) system. In this regard, it can be observed that, for a precise establishment of the intercompatibility of different cultivars, besides evaluating pollen germination, it is also necessary to evaluate the occurrence of fertilization, and this was not done (Saumitou‐Laprade, Vernet, Vekemans, Castric et al., 2017). Bradley and Griggs (1963) and Ouksili (1983) examined olive crosses with pollen germination tests on different pairs of varieties, in different conditions, and they never concluded on SI based on pollen tests alone, but they also examined ovule fertilization by pollen tubes. More recently, Seifi et al. (2011), Seifi, Guerin, Kaiser, and Sedgley (2015) and Vuletin Selak, Cuevas, Goreta Ban, and Perica (2014) examined crosses between other varieties and concluded that observations of pollen tube progression are easy, but whether one or two pollen tubes reach one of the ovules is not so easy to determine in practice. Thus, the method remains delicate to routinely ascertain whether fertilization has occurred, and the observation of fertilization is a key step to establishing compatibility or incompatibility.

Some of the results obtained by (Saumitou‐Laprade, Vernet, Vekemans, Billiard et al. (2017); Saumitou‐Laprade, Vernet, Vekemans, Castric et al. (2017)) are in contrast to results obtained in other studies (Alagna et al., 2016; Bini, 1984; Collani, 2012; Collani et al., 2012; Cuevas, Rallo, & Rapoport, 1994; Morettini et al., 1972; Sharma et al., 1976; Ugrinovic & Stampar, 1996). As reported in the introduction, Saumitou‐Laprade, Vernet, Vekemans, Castric et al. (2017) explained discrepancies between their results and other studies by referring to (i) “the vast uncertainty around the genetic identity of vernacular varieties,” (ii) “the massive risk of contamination associated with commonly used pollination protocols,” and (iii) “the importance of checking for stigma receptivity in controlled crosses.” Obviously these are real potential problems. However, in the previous paragraphs we have seen that these problems are usually not substantial if experiments are carried out with all the necessary precautions. Nevertheless, we would like to discuss these discrepancies further. We will do this using results regarding two well‐known Italian cultivars, namely Frantoio and Leccino, for which some studies are available and so it is also possible to evaluate the consistency of the results obtained in different experiments by different authors. These two cultivars were evaluated using the bagging technique. The results showed that the cultivar Leccino is self‐incompatible and the cultivar Frantoio is self‐compatible (Farinelli et al., 2008b; Morettini et al., 1972; Pannelli et al., 2000; Sharma et al., 1976; Spinardi & Bassi, 2012; Tombesi et al., 1982), whereas Saumitou‐Laprade, Vernet, Vekemans, Billiard et al. (2017), using pollen germination tests, asserted that both of them are self‐incompatible. If this last result is correct, it means that in all other studies, the results were affected by significant pollen contamination. It is difficult for us to accept this because if pollen contamination had occurred, it should have happened in both cultivars, and it seems very strange that, in different studies carried out in different environments and years, it only occurred in Frantoio and never in Leccino! Moreover, Frantoio resulted self‐compatible and Leccino self‐incompatible also in a study where pollen tests were used along with molecular analysis (Collani, 2012; Collani et al., 2012). In these studies, carried out by several of the authors of (Saumitou‐Laprade, Vernet, Vekemans, Billiard et al. (2017); Saumitou‐Laprade, Vernet, Vekemans, Castric et al. (2017)), the cultivar Frantoio showed several pollen tubes growing through the style transmitting tissue, while, in contrast, in Leccino, no pollen tubes penetrated the stigma surface. Can this be explained again with pollen contamination? Does pollen contamination always occur in Frantoio and not in the other cultivar? Moreover, in the same paper genes known to play a crucial role in SSI were “differentially expressed in flower organs of self‐compatible (cv Frantoio) and self‐incompatible (cv Leccino) genotypes” (Collani et al., 2012). In addition, in a more recent work, the analysis of the genes that were differentially expressed between Frantoio (self‐compatible) and Leccino (self‐incompatible) at anthesis enabled identification of the candidate genes that may be involved in pollen–pistil interactions (Alagna et al., 2016). It is surprising that these discrepancies were not discussed in Saumitou‐Laprade, Vernet, Vekemans, Castric et al. (2017), especially as several authors of this paper are also authors of Collani et al. (2012) and Alagna et al. (2016).

## 6 POLLEN GERMINATION VERSUS BAG METHOD

We agree with Saumitou‐Laprade, Vernet, Vekemans, Castric et al. (2017) that the mating system of the olive tree is still a controversial issue in the literature. We also agree with Saumitou‐Laprade, Vernet, Vekemans, Castric et al. (2017) on the importance of using controls for pollen contamination with paternity analyses and of using positive pollination controls for stigma receptivity. Undoubtedly, these techniques are useful for a better understanding of the mating system in olive. However, as a key step to establishing compatibility or incompatibility, we think that the data of Saumitou‐Laprade et al. about pollen germination should be integrated with observations on effective fertilization. Moreover, it could be useful to clarify discrepancies of the results of pollen tests obtained in different studies (Collani et al., 2012; Saumitou‐Laprade, Vernet, Vekemans, Castric et al. (2017)). Maybe, both the status of the trees from which flowers are collected, which could also have been affected by seasonal/climatic patterns, and the experimental conditions could have played a role in causing variability in the results. This was also presumed in other studies (Bradley et al., 1961; Fernandez‐Escobar, Ortiz ‐Urquiza, Prado, & Rapoport, 2008; Griggs et al., 1975; Guerin & Sedgley, 2007; Lavee & Datt, 1978; Martin, 1990; Perica et al., 2001; Rallo et al., 1990; Rapoport, 2014; Seifi et al., 2015; Spinardi & Bassi, 2012), and this has to be established for a routine use of pollen tests.

When the bagging technique is used, most attention must be paid in order to minimize possible problems due to uncertainty around the genetic identity of varieties, risk of pollen contamination, and poor stigma receptivity. However, we have shown that errors due to these factors are sometimes present, but do not seem to be the rule. On the other hand, it is also important to note that with the techniques used by Saumitou‐Laprade, Vernet, Vekemans, Castric et al. (2017), occurrence of contamination during pollen germination tests and reduction in pollen germination caused by pollen storage cannot be excluded (Shu‐Biao, Collins, & Sedgley, 2002).

As far as the bagging method is concerned, it would also be important to standardize the methodology to evaluate fruit set. Indeed, Musho (1977) and Ouksili (1983), and later Farinelli et al. (2006), standardized fruit for 100 hermaphroditic flowers, whereas Moutier et al. (2006) referred fruit set to 100 inflorescences, and Saumitou‐Laprade, Vernet, Vekemans, Billiard et al. (2017) counted the fruits, but did not provide data on the inflorescence architecture of each variety and on standardized fruit set, which does not allow the results of the different studies to be compared. In this regard, particular importance should be given to the number of hermaphroditic flowers.

## 7 CONCLUSION

We can conclude that, considering the contrasting conclusions drawn by different research groups (Breton et al., 2014; Saumitou‐Laprade, Vernet, Vekemans, Billiard et al. (2017)), further studies are needed for a definitive definition of the mating system in olive. This is in agreement with Lavee, Taryan, Levin, and Haskal (2002), who suggested that multiple origins of the domesticated *Olea europaea* have resulted in a complex system controlling self‐incompatibility, which is not easy to understand. Therefore, it could be helpful to use all the approaches in an integrated way in order to maximize the reliability of the information which can be drawn. As done by Saumitou‐Laprade, Vernet, Vekemans, Castric et al. (2017), we also encourage researchers to assess reproducibility of output data using all the approaches of their experimental crosses.

## CONFLICT OF INTEREST

None declared.
